# Review of Ribosome Interactions with SARS-CoV-2 and COVID-19 mRNA Vaccine

**DOI:** 10.3390/life12010057

**Published:** 2022-01-01

**Authors:** Jiao Wei, Aimin Hui

**Affiliations:** Shanghai Fosun Pharmaceutical Industrial Development, Co., Ltd., 1289 Yishan Road, Shanghai 200233, China; weijiao@fosunpharma.com

**Keywords:** ribosome, SARS-CoV-2, COVID-19 mRNA vaccines

## Abstract

Severe Acute Respiratory Syndrome Coronavirus 2 (SARS-CoV-2) is the causing pathogen of the unprecedented global Coronavirus Disease 19 (COVID-19) pandemic. Upon infection, the virus manipulates host cellular machinery and ribosomes to synthesize its own proteins for successful replication and to facilitate further infection. SARS-CoV-2 executes a multi-faceted hijacking of the host mRNA translation and cellular protein synthesis. Viral nonstructural proteins (NSPs) interact with a range of different ribosomal states and interfere with mRNA translation. Concurrent mutations on NSPs and spike proteins contribute to the epidemiological success of variants of concern (VOCs). The interactions between ribosomes and SARS-CoV-2 represent attractive targets for the development of antiviral therapeutics and vaccines. Recently approved COVID-19 mRNA vaccines also utilize the cellular machinery, to produce antigens and trigger immune responses. The design features of the mRNA vaccines are critical to efficient mRNA translation in ribosomes, and are directly related to the vaccine’s efficacy, safety, and immunogenicity. This review describes recent knowledge of how the SARS-CoV-2 virus’ genomic characteristics interfere with ribosomal function and mRNA translation. In addition, we discuss the current learning of the design features of mRNA vaccines and their impacts on translational activity in ribosomes. The understanding of ribosomal interactions with the virus and mRNA vaccines offers the foundation for antiviral therapeutic discovery and continuous mRNA vaccine optimization to lower the dose, to increase durability and/or to reduce adverse effects.

## 1. Introduction

SARS-CoV-2 is the causing pathogen of the COVID-19 pandemic that has resulted in more than 250 million cases and 5 million deaths [1,2,3]. Viruses employ the host cellular translation machinery to synthesize their own proteins. Consequently, they have developed specialized mechanisms to commandeer the host machinery. SARS-CoV-2 uses a multipronged procedure to manipulate host cellular machinery, to reduce global protein translation and engage cellular resources in order to regulate their own protein production. As the factory for protein synthesis in human cells, ribosomes play a critical role in infection and human antiviral responses.

Each human ribosome consists of 2 unequal sized subunits; one is a 40S small subunit and the other one is a large subunit (60S) [4]. The 40S small subunit is the decoding site that consists of the 18S ribosomal RNA (rRNA) and 33 proteins. At the decoding site, sequence information of the messenger RNA (mRNA) is translated into a protein sequence. The 60S subunit is the peptidyl transferase center that harbors 28S, 5.8S, and 5S rRNAs, along with 47 proteins [4]. The peptidyl transferase catalyzes the peptide bond formation between amino acids of the nascent protein at the 60S subunit [5]. The protein synthesis process begins with translation initiation, a highly ordered process that regulates mRNA translation, which is followed by elongation, during which a newly translated amino acid is added to the growing protein chain. The process is terminated when the ribosome completes the translation with a stop codon. The protein synthesis process is an energy-intensive and tightly regulated cellular process, with ribosome recycling being the last major step of this process [6].

## 2. SARS-CoV-2 Interfere with Ribosome mRNA Translation

**Figure 1:**
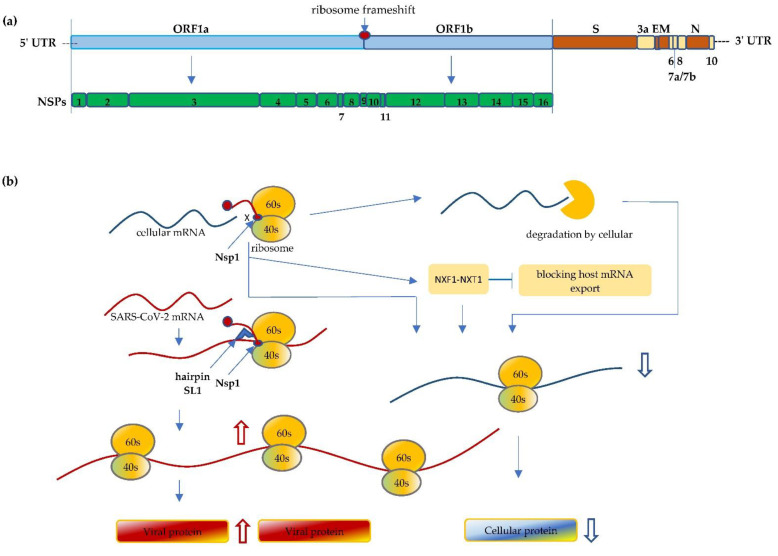
(**a**) Schematic illustration of the SARS-CoV-2 genome. The size of the coronavirus genome is approximately 30 kb in length and comprises a 5′-cap, 5′ untranslated region (5′ UTR), ORF1a/ORF1b, spike (S), envelope (E), membrane (M), nucleocapsid (N), and 3′ UTR-poly(A) tail. The first ORF comprises of an approximated 2/3 of the genome that encodes the nonstructural proteins (Nsp1 to Nsp16). (**b**) Nsp1 of SARS-CoV-2 binds to the 40S mRNA and block the mRNA entrance channel. Following viral infection, SARS-CoV-2 performs a multifaceted hijack on host machinery, including blocking the mRNA entry channel, accelerating host mRNA degradation, and inhibiting host mRNA nucleus export. Furthermore, Nsp1 interacts with 5′ UTR of SARS-CoV-2 and facilitates the translation of its own protein, resulting in viral replication and protein accumulation, and inhibiting anti-viral immune responses.

The SARS-CoV-2 is a novel beta-coronavirus, a category that also includes two already known virulent coronaviruses, namely SARS-CoV-1 and MERS-CoV, that have resulted in serious outbreaks in 2002 and 2012, respectively [7]. SARS-CoV-2 is an enveloped, positive-sense and single-stranded RNA virus; its genome is approximately 30 kb in length. The SARS-CoV-2 genome comprises a 5′-cap, 5′ untranslated region (5′ UTR), followed by -replicase (ORF1a/ORF1b)-Spike (S)-Envelope (E)-Membrane (M)-Nucleocapsid (N)-3′ UTR-poly(A) tail [8] (Figure 1). Though it shares more than 80% homology with SARS-CoV-1 and ~50% with MERS-CoV, the mortality rates of these infections are slightly different, ranging from 15% for SARS-CoV-1 and 34.4–37% for MERS-CoV, to around 2–13% for SARS-CoV-2 [1,9,10,11,12].

At the 5′ end of SARS-CoV-2 genome, it starts with two large overlapping ORFs (ORF1a and ORF1b) representing two-thirds of its genome, with the other one-third encoding the structural proteins and accessory proteins [13,14]. Upon entering host cells, the ORF1a and 1b of the viral genomic RNA are translated and continually produce polyprotein, which is then cleaved into functional NSPs. These NSPs play pivotal roles, like evading the host immune system. Multiple NSPs interact with each other or form complex structures to modify cellular conditions for efficient mRNA translation and viral replication [15]. The 440–500 kDa Polyprotein 1a (pp1a) is first translated from ORF1a and then cleaved from Nsp1 to Nsp11. Between ORF1a and 1b, the programmed ribosomal -1 frameshift (PRF) of the reading frame immediately takes place preceding the stop codon of ORF1a, which enables the downstream translation of pp1ab from ORF1b. The pp1ab is subsequently processed into functional Nsp1 through Nsp16 [16].

The PRF, a well-preserved process among coronaviruses, is essential for functioning translation of NSPs. An efficient PRF engages a conserved slippery sequence (U_UUA_AAC) that changes the reading frame to UUU_AAA_C after the frameshifting [17]. When a ribosome approaches the slippery site during translation, a stimulatory RNA folds into a stable pseudoknot structure that slows down translation and promotes the PRF [17]. Importantly, Nsp12 and downstream NSPs that are involved in RNA capping, modification, and proofreading, rely on the PRF process as they are translated after the frameshifting [17]. It has been reported that multiple factors, including, for example, the position of the ORF1a stop codon, and interactions between ribosomal tunnels and RNA elements, including pseudoknot and nascent chain, modulate the optimum efficiency of frameshifting [17]. In addition to RNA regulation, the zinc-finger antiviral protein (ZAP-s) has been observed to interact with viral RNA and interfere with PRF [18]. PRF is one of the crucial steps in ribosome translation of virus genomes and viral replication, and thus presents a viable potential target for antiviral intervention therapeutics [17,19,20].

Among the 16 NSPs, Nsp1 is one of the first functional coronaviral nonstructural proteins translated in infected cells. Nsp1 is a protein comprised of 180 amino acids that targets cellular processes to inhibit translation, triggers host mRNA cleavage and decay, and down-regulates type I interferon (IFN) response [21,22,23,24]. NSP1 is a major virulence factor that is essential for viral replication, and is thus emphasized here [15,19,22,25].

Known as the host shutoff factor, Nsp1 efficiently interacts with an array of different ribosomal states, resulting in a shutdown of host protein production [15,22]. The 40S subunit plays a critical function in the highly regulated translation initiation process, in which it binds initiation factors to form 43S pre-initiation complexes and facilitates scanning of the 5′ UTR to the AUG start codon [26]. It has been reported that Nsp1 manipulates the ribosome at the translation initiation step and stalls canonical mRNA translation directly through binds to the ribosomes’ 40S subunit, the 43S pre-initiation complex, and the 80S non-translating ribosome [15,21,22]. The C-terminal domain of the Nsp1 protein folds into two helices that physically block mRNA accommodation at the entrance channel and shut down host mRNA translation [15]. Mutants of the C-terminal domain led to the abolishment of the Nsp1’s ability to bind 40S subunits, indicating that the helices area, specifically aa 154–165 and 171–179, are crucial for Nsp1–ribosome interactions [15,25].

In addition to inhibition of the host mRNA translation, SARS-CoV-2 has developed manifold instruments, including, for example, the degradation of host mRNA and the blocking of host mRNA export. Nsp1 facilitates accelerated host mRNA degradation, through mRNA endonucleolytic cleavage in the 5′-UTR of the host mRNA. In particular, the R124 and K125 aa sites of the Nsp1 protein play a pivotal role in this cleavage [25]. The accelerated degradation of cytosolic cellular mRNAs is a significant part of remodeling the mRNA pool, facilitating the viral takeover of the mRNA pool in infected cells [14,27,28]. Furthermore, Nsp1 binds to the host mRNA export factor to interact with the NXF1-NXT1 receptor resulting in the inhibition of mRNA nuclear export. As a result, host mRNA is retained in the nucleus [24] (Figure 1).

While shutting off host mRNA translation, SARS-CoV-2 orchestrates its own viral translation without inhibition under the ribosome blockade condition, which is achieved through the highly ordered process by multifunctional NSPs. Despite the Nsp1 bound on the ribosome, Nsp1 of SARS-CoV-2 can recognize and accommodate its own mRNA and proceed to initiate the translation. The 5′ UTR of SARS-CoV-2 forms a unique cis-acting RNA hairpin SL1 structure that plays a prominent role in this evasion. The SL1 structure interacts with Nsp1, which frees the mRNA accommodation channel and promotes viral translation [28]. It has been reported that the binding affinity between Nsp1 and SL1 is relatively high, which facilitates the recruitment of the 40S ribosomal subunit and favors the viral translation [29]. In addition, the viral mRNA is protected from endonucleolytic mRNA cleavage and degradation. The interaction of Nsp1 and 5′ UTR of viral RNA can also contribute the resistant to the cleavage [29]. Another mechanism is the interaction between Nsp10, Nsp14, and Nsp16, which forms a complex that facilitates SARS-CoV-2 mRNA capping and proof-reading, providing a significant contribution in protecting SARS-CoV-2 mRNA from accelerated degradation [13]. Through a collective mechanism, Nsp1 of SARS-CoV-2 is vital for viral replication as it not only hampers the translation of cellular transcripts, shutting off host protein production, but also has the ability to recruit the ribosome in order to efficiently translate the viral mRNA to allow the expression of viral genes [15].

Type I interferon induction and innate interferon response represent one of the major innate antiviral host defenses against viral infections [30]. SARS-CoV-2 efficiently suppresses the IFN-I signaling, likely mediated by the inhibition of STAT1 and STAT2, resulting in lack of efficient IFN-dependent antiviral innate immune responses. In addition to the shutting-off of host mRNA translation, the inhibition of INF-I antiviral responses leads to higher viral replication, viral protein accumulation, and pathogenesis [30]. Collectively, a fully functional Nsp1 is necessary for virulence; thus, the targeting of Nsp1 proteins and the Nsp1–ribosome interactions presents an attractive therapeutic opportunity for future studies [13,22,28].

## 3. Mutations Impact Replication and Virulence

Although SARS-CoV-2 has proof-reading processes, mutations arise naturally during viral replication, which causes new variants to form. Since late 2020, several novel variants have been named SARS-CoV-2 variants of concern/interest (VOC/I) due to their greater risk of enhanced transmissibility, pathogenicity and/or ability to evade host response [31]. The Alpha (B.1.1.7) variant, first detected in England in September 2020, appears to have a higher reproduction number and transmits more efficiently from person to person [32]. The Beta (B.1.351) variants, first reported in South America, and Gamma (P.1) variants share some of the same genetic changes that are associated with the increased transmission, and higher viral load. It is reported that these mutations lead to immune escape from neutralizing antibodies [33,34]. The Delta (B.1.617.2) variant is believed to be highly transmissible with more than twice as the original strain of SARS-CoV-2 [35]. Since first appearing in India in late 2020, it has spread worldwide and became the dominant variant of SARS-CoV-2 virus in the U.S. in late 2021 [36]. The recent emergence of Omicron has raised significant concern due to its extensive mutations, including more than 30 mutations on the Spike protein [37].

**Figure 2:**
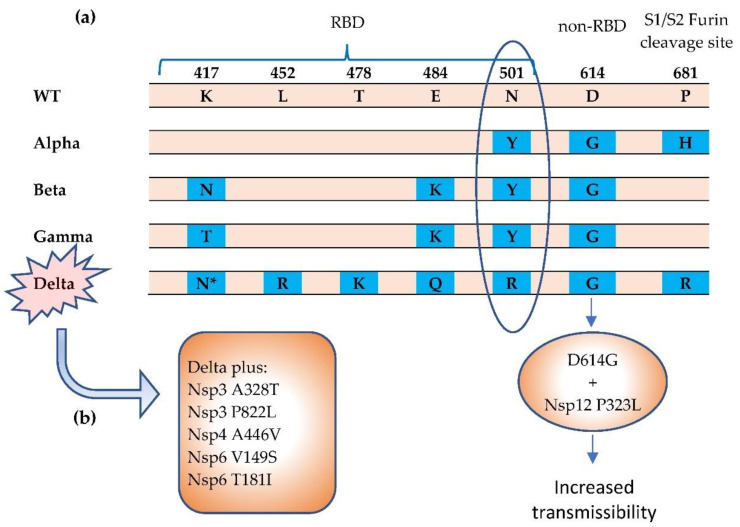
The Alpha, Beta, Gamma, and Delta variants have been termed SARS-CoV-2 COVs. (**a**) Notable mutations in spike protein [38,39,40]. The N501Y mutation results in greater affinity for ACE2 receptor, which can increase transmissibility. (**b**) The Delta variant carries a more diverse repertoire of mutations [43,44]. The Delta Plus variant carries increased mutations in NSPs. * = K417N. The K417N mutation is significantly more prevalent in the Delta Plus (AY.1 or B.1.617.2.1) variant than in the Delta (B.1.617.2) variant. RBD = receptor binding domain.

The virus is able to mutate in a dangerous and clever way to become structurally more infectious or cause more severe disease, in which multiple mechanisms may be operating. The major VOCs have shared mutations in the spike protein of SARS-CoV-2 genome, mostly on the S1 subunit, which is the unit that possesses the receptor-binding domain (RBD) that binds to cellular receptor ACE2 through six key amino acid residues [38] (Figure 2). N501 on the RBD domain is one of these amino acids that has a specific interaction with ACE2 receptor. Observed in three variants (Alpha, Beta, and Gamma), the N501Y mutation raises considerable concern due to its greater ACE2 binding affinity and enhanced transmissibility [39,40]. The Beta variant carries K417N and E484K mutations while Gamma shares E484K, along with the mutation of K417 to K417T [40]. These S1 mutations increase the binding affinity to the ACE2 receptor, thereby possibly enhancing transmissibility, which can affect disease severity and clinical outcomes [38].

The Delta variant has picked up new mutations, including multiple mutations (L452R, E484Q, T478K) in the S1 subunit, resulting in a viral load which is more than 1000 times higher than the original strain infections [35]. The Delta variant is broken into a few subtypes that are being classified as Delta Plus, which has drawn greater attention recently [41]. In addition to the Delta mutations, the Delta-AY.1 variant carries K417N mutation that can interact with N501Y, which can increase the binding affinity with ACE2 receptor and possibly reduce neutralizing antibody susceptibility [41]. D614G, a non-RBD site mutation on spike proteins, represented in more than 90% of prevalent variants, has become the most predominant mutation since it first emerged in early 2020 [38]. It is reported that the D614G is related to increase spike density and cell entry, but does not directly increase the ACE2 receptor binding affinity or reduce the susceptibility of neutralizing antibodies for the virion [42].

Although our understanding of the functional consequences of spike mutations is rapidly expanding, the mutations in NSPs, 3′ structural proteins, and accessory proteins are relatively under-investigated despite playing a significant role in virus translation, replication, and host immune suppression.

NSP1 is a highly conserved protein with few mutations that have been identified [25]. The two helices structure, including in particular the KH motif K164 and H165 in C-terminal of the SARS-CoV-2 Nsp1, is crucial for ribosome binding and the inhibition of translation initiation. [15]. However, it is interesting that a deletion hotspot at the 500–532 locus of the Nsp1 N-terminus coding region has been identified, which has been detected in 37 countries [45]. Although the Nsp1 mutants retain the binding ability to the 40S subunit, it significantly suppresses IFN-I response [46]. The deletion of 500–532 is an in-frame deletion, which is likely important to Nsp1 N-terminal structure, as it can affect its function related to suppressing innate immune factors [30,45].

Several hot-spot mutations have been identified on NSPs. For example, L37F on Nsp6 and P323L on Nsp12 emerged in January 2020, followed by G15S on Nsp5, T428I on Nsp3, and T85I and I120F on Nsp2, which emerged in March 2020 [47]. In addition to NSPs mutations, one of the major mutations is Q57H on ORF3a, which appeared concurrently with D614G and Nsp2 T85I in early 2020, and became one of the most frequent sub-haplogroups in the U.S. (~45%) and worldwide (~36%) [48]. The T85I mutation on Nsp2 involves a change from polar and uncharged threonine(T) to isoleucine (I), an uncharged, non-polar amino acid, which can affect protein structure and is possibly linked to infectivity and virulence [49]. The concurrent mutations of Spike D614G, ORF3a Q57H, and Nsp2 T85I can synergistically enhance the viral transmissibility that correlates with the dominant spreading variants [48].

In addition to the signature K417N mutation, the Delta Plus has additional mutations in NSPs that affects the virulence [43]. The Nsp3 A328T, Nsp3 P822L, Nsp4 A446V, Np6 V149S, and Nsp6 T181I mutations appear in more than 50% of Delta Plus mutations, but are not present or are at a low percentage in Delta [43]. Spike mutation W258L mutation, which exists in ~40% in the Delta Plus variant, strongly correlates with other Spike mutations (G142D, T95I) and the Nsp4 A446V mutation [43]. Furthermore, Nsp4 A446V highly correlates with the D950N mutation on the spike protein, and is approximately 90% present, currently [43].

Nsp12, together with Nsp7 and Nsp8, represents the RNA-dependent RNA polymerase (RdRp) complex that is essential for SARS-CoV-2 replication and survival [50]. Nsp12 is structurally conserved; its structure and the potential binding site and binding activities have been extensively investigated [44,51,52,53]. For example, catalytic motifs (A-E) at the palm domain have been considered potential binding sites, due to their structurally conserved attributes and importance for enzymatic functions [52]. The interface domain mutation P323L on Nsp12, which emerged concurrently with D614G, has become one of the dominant mutations worldwide since it was first emerged in early 2020 [49]. It is reported that the mutation from proline (P) to leucine (L) will possibly alter the local structural flexibility or viral proofreading ability [44,47,54]. In addition, the P323L mutation can be associated with enhanced Nsp8 interaction, which is important for RdRp enzyme activity. Interestingly, it is the striking evolution of D614G with the P323L combination, but not G614 or L323 stand-alone mutation, that becomes the epidemiologically triumphant mutation of the present VOC and displays supremacy over original D614 and P323 [44].

In addition to spike mutations, the combined effect of the mutations on structural and nonstructural proteins, particularly concurrent mutations in NSPs and the spike protein, necessitates further research to gain insights into virulence associated with the variants, which is imperative for the development of therapeutics and vaccines.

## 4. Molecule Design Impacts Ribosome Vaccine mRNA Translation

During the COVID-19 pandemic, scientists spent the past year developing vaccines and treatments to lessen the disease’s damage. The vaccine is a critical tool to help stop the pandemic. To date, two COVID-19 mRNA vaccines (BNT162b2 and mRNA-1273) are authorized and distributed worldwide [55,56]. In principle, an mRNA vaccine comprises synthetic mRNA molecules coded with the sequence of immunogen, that direct the cell machine, ribosomes, to produce vaccine protein antigens and generate an immune response. Once the vaccine is delivered into the cells, the ribosomes translate the mRNA vaccine sequence and produce the antigen, which is the spike protein of SARS-CoV-2 for the COVID-19 vaccine. The produced spike protein then triggers the immune response, including the production of antibodies and the cellular immune response [57].

Both BNT162b2 and mRNA-1273 utilize modified mRNA technology to code an optimized version of the spike protein sequence. The nucleoside-modified mRNA is encapsulated in a lipid nanoparticle with different lipids and formulations. Through intramuscular injection, BNT162b2 is administered in 21 days apart with a 30 µg dose; while mRNA-1273 is given in two 100 µg doses with 28 days in between each dose. Both BNT162b2 and mRNA-1273 have been shown to be safe and highly effective with 95% and 94.1% efficacy, respectively, in large Phase 3 double-blind clinical trials [58,59,60,61].

**Figure 3:**
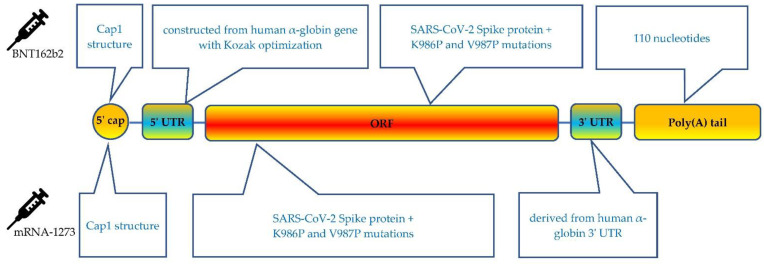
Schematic presentation of the BNT162b2 and mRNA-1273 COVID-19 mRNA vaccine.

The mRNA vaccines contain 5 essential components, including 5′ cap, 5′ UTR, an open reading frame (ORF) that encodes the antigen, 3′ UTRs and a 3′ poly(A) tail [62] (Figure 3). Although both mRNA vaccines share the same antigen sequence of SARS-CoV-2 spike protein, each vaccine involves many different types of optimizations. In addition to the ongoing efforts for optimizing the delivery technology, optimizing the technical basis of the design features is critical in regulating the interaction of the vaccine and ribosomes can reduce dosage to be injected, lead to more efficient immunization, and improve safety [57].

Each functional component in the mRNA vaccine can be independently optimized. It is well known that the 5′ cap structure of the mRNA molecule is the essence for the efficient translation of mRNA on the ribosome. The 5′ cap protects mRNA from degradation and interacts with the eukaryotic initiation factor (eIF) 4E to recruit 40S ribosome subunits and promotes the translation initiation complex of ribosomes [63,64,65]. In addition, it plays a prominent role in antigen production and prevents unintended immune responses by preventing recognition by cytosolic sensors of viral RNA [66,67]. In eukaryotes, a cap structure, either Cap 0 [m7G(5′)pppN1pN2p] or Cap 1 [m7G(5′)pppN1mpNp], is associated with the 5′ end of the mRNA [64,65]. The addition of the 5′ cap in mRNA vaccines can be achieved either co-transcriptionally, in which the cap is attached when the rest of the mRNA is assembled, or post-transcriptionally, in an additional process step after transcription [68]. Capping of BNT162b2 uses a trinucleotide Cap 1 analogue that is co-transcriptionally produced, while for the mRNA-1273 vaccine, the capping process is performed using a vaccinia capping enzyme post transcription [62,69].

The 5′ and 3′ UTR regions are engaged in the half-life, localization, translation efficacy, and recruiting of mRNAs to the ribosomes [64,70,71]. Therefore, the molecular design and optimization of the UTRs are critical for mRNA vaccine to ensure efficient antigen production and immune responses [72]. The highly expressed and naturally existing UTRs from human genes are often advantaged and employed for mRNA vaccines [73]. The 5′ UTR of BNT162b2 is constructed from the human α-globin gene using Kozak sequence optimization [74]. Similar approaches were naturally followed when designing the 3′ UTR, by incorporating regulatory elements for stability from human α-globin and β-globin [74,75]. The mRNA-1273 has a 110-nt 3′ UTR of the human α--globin gene (HBA1) inserted into its 3′ UTR region. [74].

There is a growing body of knowledge on alternative UTR sequences, such alteration in the UTRs, which can affect the functionality of the ribosomes [76]. For example, the length and structures of the 5′ and 3′ UTR, and regulatory components in the UTR sequences alter mRNA translation characteristics and protein synthesis [77]. Length is critical because the 5′ UTR serves as the ribosome landing zone and physically provides “lead-in”. In addition, the nucleotide composition of A, C, G, and U are considered important factors that would impact structure and mRNA stability. High GC content is likely to induce secondary structural formation and increased mRNA stability, while an AU rich region is associated with translation regulation [73,78]. In addition, microRNA binding sites should be avoided as it can bind to target sites within 5′ UTR and interfere with the ribosomal scanning [79]. The length of the 3′ UTR can be optimized, for example, as mRNAs with longer 3′ UTRs is commonly associated with shorter half-life, whereas shorter 3′ UTRs are associated with decreased translation efficiency [80]. The secondary structures can prevent ribosomes from skipping the stop codon and benefit protein production [81,82].

The mRNA sequence encodes trimerized SARS-CoV-2 S protein with K986P and V987P mutation optimizations for prefusion stabilization of the translated spike protein [83,84]. As mammalian host cells attack unmodified exogenous RNA as it activates innate immune response [85]. The modifications of N1-methyl-pseudouridine (N1m) lead to increased stability and low immunogenicity. Incorporation of N1m nucleotides facilitates ribosome loading and increases its density on mRNA, resulting in the alteration of translation dynamics. [63]. In COVID-19 mRNA vaccines, uridine bases are replaced with N1m to improve safety and translation efficacy [69,86].

The very end of mRNA is polyadenylated. The proper length and properties of the poly(A) tail are important for mRNA translation. In general, a 100–150 bp poly(A) tail is considered a sufficiently long enough tail to interact with poly(A) binding proteins, which is necessary for translation initiation [87,88]. In addition, the poly(A) tail is crucial for protection of the cap from degradation by de-capping enzymes [89]. The poly(A) tail of the BNT162b2 vaccine includes 30 A’s and 70A’s with a “10 nucleotide linker” (GCAUAUGACU) in between [90].

## 5. COVID-19 Vaccination and Boosters

**Figure 4:**
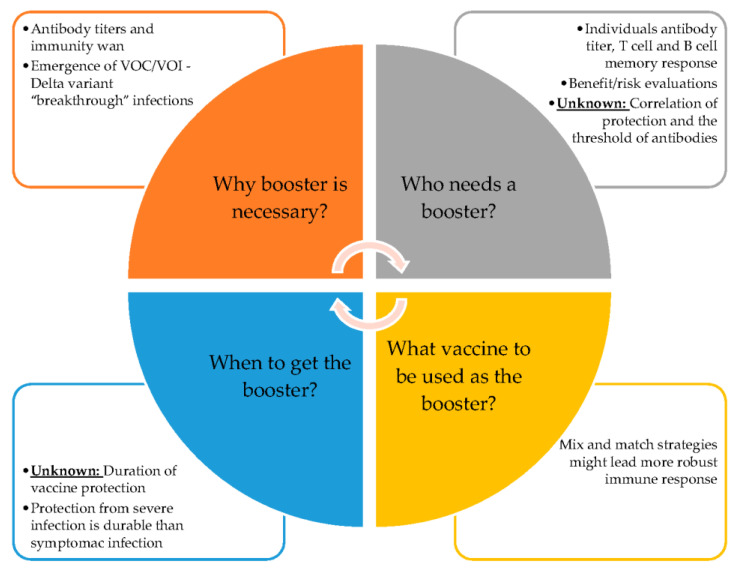
The known and unknown of COVID-19 vaccine boosters.

Upon vaccination, the ribosomes make copies of the spike protein and trigger the immune response. The neutralizing antibodies’ levels, as well as binding antibodies, are considered important predictors of levels of vaccine protection [91,92]. However, the vaccine antibody titers and T-cell titers will decay overtime, and the durability of protection of COVID-19 vaccines remains unknown. The issue of booster shots is now on the horizon amid questions about the “breakthrough” infections among the fully vaccinated with the primary series, particularly with the spreading of the Delta and Omicron variants that might be able to evade COVID-19 vaccine antibodies [92,93]. The important question is whether or not a booster is necessary, for who and when boosters might be needed, as well as whether boosters can protect against VOCs and other potential new variants (Figure 4).

In clinical studies, both BNT162b2 and mRNA-1273 elicited dose-dependent SARS-CoV-2 antibody responses. At the 30 µg dose level of BNT162b2, the 50% neutralizing geometric mean titers (GMT) ranged from 149 to 361, approximately 1.5 to 3.8 times the GMT of the convalescent serum, at 7 or 14 days after the second shot. The young adult (18–55 years of age) generates a higher neutralizing response than the 65 to 86 age group [58]. At the 100-μg dose of mRNA-1273, the antibodies levels remain detectable through the 6 months period after the second injection; however, it peaked at 14 days post second dose vaccination and decreased overtime [94]. Utilizing live-virus neutralization assay, the GMT ranged from 131 to 406, with the younger age group (18–55 years of age) at the highest [94]. Both mRNA vaccines have reported common but mild reactions at the local injection site. Systemically, fatigue, headaches, muscle pains, and fevers are some of the most frequently reported adverse actions [60,61,95].

The BNT162b2 vaccine efficacy against COVID-19 is waned slightly over time after 6 months of study follow-up, during the pre-Delta period. With the peak of 96% within 2 months from 7 days after the second injection, the vaccine efficacy gradually declines to 83% at between 4 to 6 months after the second dose, with a decline of approximately 6% every 2 months [96]. The Delta variant became widespread globally in June 2021, and currently is the dominant variant [97]. Although the efficacy of the currently approved vaccine has slightly decreased with the Delta variant, data has shown that it still provides strong protection. The estimated effectiveness again the Delta variant is between 85 to 90 percent after two doses of the BNT162b2 [98].

Some people with underlying diseases or medical conditions have a reduced ability to fight COVID-19 or other infections. In addition, certain immunocompromised individuals, specifically solid organ transplant recipients, develop lower antibody responses compared to healthy individuals. In this case, a third dose is necessary to boost their level of immunity to the coronavirus and protect them from serious diseases [99]. A double-blind study has shown that the third dose of mRNA-1273 is safe and can induce increased immunogenicity response [100].

It is reported that the heterologous vaccination, for example, the ChAdOx1 nCoV-19 prime with a BNT162b2 or mRNA-1273 booster, has significantly greater effectiveness than the homologous vaccination [101]. These “mix and match” vaccinations can be an effective alternative strategy to increase population immunity against the impact of COVID-19 variants [101]. Recently, both BNT162b2 and mRNA-1273 boosters have been authorized for use for adults that are 18 years of age and older [102]. The booster dose has been shown to increase the vaccine protection of variants, and the BNT162b2 booster has shown to decrease the viral load of the Delta variant [103,104].

Vaccination in children is incredibly important, as currently, infections in children account for approximately a quarter of COVID-19 cases in the U.S. [105]. In addition to the authorization of the usage of BNT162b2 in children who are 12 years and older, the low dose of BNT162b2 has showed to be safe and effective for children in 5 to 12 years of age [106,107]. Further, the 25 µg low dose of mRNA-1273 has induced durable antibodies, CD4+, and CD8+ T cells, which can offer a potential for vaccination in children [108]. Although many concerns around limited testing and the long-term safety of the novel mRNA platform in children have been raised, the benefits-over-risks consideration serves as the golden rule for this important decision.

## 6. Future Prospects

Understanding the biological basis of SARS-CoV-2 infection is the key to developing safe and effective treatments and vaccination strategies to tackle this COVID-19 pandemic. The success of the COVID-19 mRNA vaccine will shape the future of drug development. With the successful development of the COVID-19 vaccine, the mRNA vaccines will become a platform approach that can develop vaccines quickly, helping to prevent the blight of infectious diseases. In addition, mRNA technology has excellent potential beyond vaccines to treat other intractable disorders, including cancer, which will have a tremendous impact in the next decade.

## Data Availability

Not applicable.
